# Colonic response to laxative ingestion as assessed by MRI differs in constipated irritable bowel syndrome compared to functional constipation

**DOI:** 10.1111/nmo.12784

**Published:** 2016-02-12

**Authors:** C. Lam, G. Chaddock, L. Marciani, C. Costigan, J. Paul, E. Cox, C. Hoad, A. Menys, S. Pritchard, K. Garsed, S. Taylor, D. Atkinson, P. Gowland, R. Spiller

**Affiliations:** ^1^ NIHR Nottingham Digestive Diseases Biomedical Research Unit and Nottingham Digestive Diseases CentreSchool of MedicineUniversity of NottinghamNottinghamUK; ^2^ Sir Peter Mansfield Imaging CentreUniversity of NottinghamNottinghamUK; ^3^ Centre for Medical ImagingDivision of MedicineUniversity College LondonLondonUK; ^4^ Royal Derby Hospitals Foundation TrustDerbyUK

**Keywords:** colon, functional constipation, irritable bowel syndrome with constipation, laxative, motility

## Abstract

**Background:**

Functional constipation (FC) and irritable bowel syndrome with constipation (IBS‐C) share many symptoms but underlying mechanisms may be different. We have developed a magnetic resonance imaging (MRI) technique to measure intestinal volumes, transit, and motility in response to a laxative, Moviprep^®^. We aim to use these biomarkers to study the pathophysiology in IBS‐C and FC.

**Methods:**

Twenty‐four FC and 24 IBS‐C were studied. Transit was assessed using the weighted average position score (WAPS) of five MRI marker pills, taken 24 h before MRI scanning. Following baseline scan, participants ingested 1 L of Moviprep^®^ followed by hourly scans. Magnetic resonance imaging parameters and bowel symptoms were scored from 0 to 4 h.

**Key Results:**

Weighted average position score for FC was 3.6 (2.5–4.2), significantly greater than IBS‐C at 2.0 (1.5–3.2), *p* = 0.01, indicating slower transit for FC. Functional constipation showed greater fasting small bowel water content, 83 (63–142) mL *vs* 39 (15–70) mL in IBS‐C, *p* < 0.01 and greater ascending colon volume (AC), 314 (101) mL *vs* 226 (71) mL in IBS‐C, *p* < 0.01. FC motility index was lower at 0.055 (0.044) compared to IBS‐C, 0.107 (0.070), *p* < 0.01. Time to first bowel movement following ingestion of Moviprep^®^ was greater for FC, being 295 (116–526) min, compared to IBS‐C at 84 (49–111) min, *p* < 0.01, and correlated with AC volume 2 h after Moviprep^®^, *r* = 0.44, *p* < 0.01. Using a cut‐off >230 min distinguishes FC from IBS‐C with low sensitivity of 55% but high specificity of 95%.

**Conclusion & Inferences:**

Our objective MRI biomarkers allow a distinction between FC and IBS‐C.

**Abbreviations**
AC: ascending colon
FC: functional constipation
HV: healthy volunteer
IBS‐C: irritable bowel syndrome with constipation
IBS: irritable bowel syndrome
MRI: magnetic resonance imaging
PEG: polyethylene glycol
PHQ12SS: Patient Health Questionnaire 12 Somatic Symptom scale
SBWC: small bowel water content
TTFBM: time to first bowel movement
VAS: visual analogue scale
WAPS: weighted average position score

**Key Messages**

- Physiology and motor function of the large bowel can be non‐invasively measured using magnetic resonance imaging.
- Using a stimulus such as Moviprep^®^, FC can be differentiated from IBS‐C by assessing the motility of AC and time to first bowel movement.
- The underlying disorder of function differs in FC and IBS‐C implying that response to treatments altering motility will differ.
- MRI can be used as a tool to clarify the underlying functional abnormality in patients with difficult and resistant constipation.
- Even without MRI, using a 1L of Moviprep^®^ as a stimulant and measuring the time to first bowel movement can assist in differentiating between FC and IBS‐C.

## Introduction

Constipation is a common condition with up to 27% of the population reporting suffering from constipation at least some of the time.^1^ The commonest complaints reported are straining to pass stool, gas, and hardness of stool followed by infrequent bowel movements. About half of the patients also complain of abdominal pain.^2^ Abdominal pain/discomfort is a key feature of irritable bowel syndrome with constipation (IBS‐C) which distinguishes it from functional constipation (FC) in which pain is either absent or not prominent.^3^ The other symptoms of these two conditions like hard stools and straining overlap extensively and if one suspends the requirement for FC patients to not have IBS, then 44% of patients with FC also meet Rome III IBS‐C criteria while 85% of IBS‐C meet the criteria for FC.^4^ However, making the distinction in some patients may be worthwhile since, as we show below, the underlying pathophysiology and response to treatments in chronic constipation and IBS‐C appear to differ in important ways.

Motility also appears to differ in the few studies available. Prolonged (24 h) ambulatory manometry recordings in FC with severe slow transit have shown reduced motility^5^ while one study using radiotelemetry showed that IBS‐C patients had normal or increased contractions, particularly in the distal quartile of colonic transit compared to both FC and healthy controls.^6^ Current methods of objective assessment of motility have significant limitations to widespread use because they are technically demanding and require expensive equipment and special expertise to operate. The need for bowel cleansing for both ambulatory manometry and rectal barostat testing significantly alters the underlying pathology and the techniques introduce many other variables including psychological distress. Furthermore, not all patients will agree to such invasive tests making the observations biased in unpredictable ways. There is therefore an unmet need for a more acceptable assessment.

We have recently developed a non‐invasive magnetic resonance imaging (MRI) technique which allows measurement of intestinal water content,^7^ colonic volumes,^8^ motility,^9^ and transit^10^ in a way acceptable to most patients.

The aim of this study was to combine these measurements to create a test of colonic function using as our standardized intervention a large dose of the osmotic laxative, Moviprep^®^ (a combination of PEG and electrolyte) to distend the whole colon and allow observation of the colonic response. We hypothesized that this response would differ in FC compared to IBS‐C.

## Materials and Methods

This was an open‐label study examining the response of the small and large intestine to acute ingestion of 1 L of polyethylene glycol (PEG) and electrolyte solution (Moviprep^®^, Norgine Pharmaceuticals Ltd, Harefield, UK). We used a virtually identical protocol to that already reported in healthy controls (HV)^11^ from which we derived our normal range. These studies were approved by the National Research Ethics Service, United Kingdom (10/H0906/50 and 11/EM/0440) and by the Medicines and Healthcare products Regulatory Agency (MHRA CTA reference number 03057/0045/001‐0002). This study was registered with the ClinicalTrials.gov (Identifier NCT01622972) and the EU clinical trials register with EudraCT number 2010‐021879‐85. There were no changes to the protocols from that published at registration. All participants gave written informed consent and the studies were carried out according to the Good Clinical Practice principles.

### Subjects

Forty‐eight (45 females, 3 males, 21–68 years old) patients with chronic constipation were recruited from gastroenterology clinics in the Nottingham University Hospital Trusts, Nottingham during March 2012 to February 2014. These comprised two groups classified according to the Rome III criteria into FC or irritable bowel syndrome with predominant constipation (IBS‐C).^3^ Twenty‐four patients had FC and 24 patients had IBS‐C. As this was a secondary referral practice, these patients had all failed at least one simple laxative in the past before entry into the study. Participants were required to stop any laxatives and medications that could affect the gut motility approximately 7 days prior to the allocated study day. Participants who had other chronic gastrointestinal illness, gastrointestinal surgery (except appendicectomy), or diabetes were excluded from the study. Other exclusion criteria were pregnancy and being unwilling to stop any medications that interfere with gastrointestinal function including opiates. All participants completed a safety questionnaire to exclude contraindications to MRI such as metal implants in the body. They also all completed the Hospital Anxiety and Depression questionnaire and Patient Health Questionnaire 12 Somatic Symptom scale (PHQ12SS) to assess psychological and somatic distress.

### Study design

The constipation patients followed the same protocol as a previous healthy volunteer (HV) study^11^ but in addition, they also had a MRI assessment of the whole gut transit time which required them to swallow five MRI marker pills (20 mm × 7 mm) at 8 am, which were imaged 24 h later at the beginning of the study day. The MRI marker pills were used to calculate a weighted average position score (WAPS) depending on their position in the bowel. This method of WAPS using the magnetic resonance imaging, which is similar in concept to the well‐validated Mayo technique using scintigraphy after a single dose of isotope marker,^12^ has been validated against the standard radio‐opaque marker test.^10^, ^13^ The patients were required to fast overnight before the study day. Following their baseline scans to assess the location of the transit markers in the gut and make baseline volume measurements (see MRI scanning protocol), they ingested 1 L of Moviprep^®^ containing 100 g PEG (mean molecular weight 3350 Da), Na^+^ 182, K^+^ 14, Cl^−^ 60, SO4^−^ 53, ascorbate 30 mEq within 60 min, before undergoing hourly MRI scans for 4 h. We were able to reduce the scanning time to 4 h as our HV results^11^ indicated that all the important responses could be observed within this time. Patients completed paper bowel symptom questionnaires and stool diaries throughout the study. The paper bowel symptom questionnaire required subjects to indicate on a 10‐cm visual analogue scale (VAS) scale the severity of each of the following symptoms: abdominal pain, bloating, abdominal distension, abdominal fullness, and nausea. They were required to complete this questionnaire every hour immediately following each MRI scan in order to correlate these symptoms with the MRI findings. They also filled out a paper stool diary recording each bowel movement using the Bristol Stool Form Scale^14^ and baseline symptoms for 1 week before the study day while they were off any medications that might affect gastrointestinal symptoms, during the study day and for 6 days after the study day. Particular note was made of the time to first defecation following ingestion of Moviprep^®^. Patients were allowed to drink water *ad libitum* 120 min after completing Moviprep ingestion.

### MRI scanning protocol

**Figure 1:**
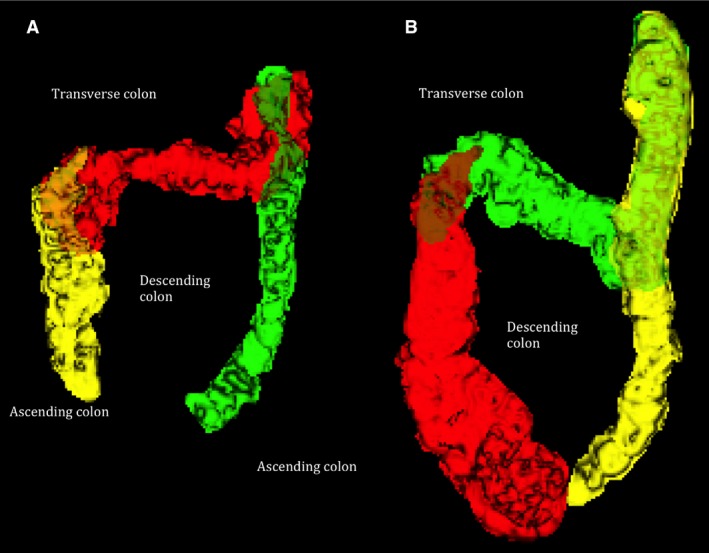
(A) Three‐dimensional segmented colon from (A) a healthy control giving a total colonic volume of 383 mL and (B) a functional constipation patient giving a total colonic volume of 1244 mL.

All MRI scans were carried out with a 1.5T Philips Achieva scanner (Philips, Best, The Netherlands), using a 16‐channel XL torso coil. All participants were scanned in a supine position for approximately 15 min and while between scans they were sat in an up‐right position in the waiting room. A turbo spin echo single shot sequence (TR/TE = 8000/320 ms, FA = 90°, FOV = 400 × 362 × 168 mm^3^, ACQ res = 1.56 × 2.90 × 7.0 mm^3^) was used to acquire T2‐weighted coronal images for measurement of small bowel water content (SBWC) as previously validated^7^ and shown to be responsive to interventions.^7^, ^15^, ^16^ This sequence gives high‐intensity signals from areas with free fluid and little signal from body tissues. Assessment of WAPS involved the acquisition of two sequences, which were obtained as coronal sections, collected at two stations with a 30‐mm overlap. Firstly, a T1‐weighted 3D Turbo Field Echo sequence was used to count and locate the number of pills remaining in the colon after 24 h of pill ingestion. Secondly, a 3D dual echo fast field echo DIXON sequence was used to confirm the location of the pills, by creating a rotating movie of the maximum intensity projection of the water‐only images as previously described.^10^ This movie allowed 3D visualization of the colon and accurate localization of the position of the pills in the uncommon event that the alternate T1‐weighted image scan was not conclusive. A coronal dual echo fast field echo sequence (TR/TE_1_/TE_2_ = 157/2.3/4.6 ms, FA = 80°, FOV = 450 × 362 × 168 mm^3^, ACQ res = 2.01 × 2.87 × 7 mm^3^) was used to assess colonic volumes.^8^ This was performed during an expiration breath hold of 13 s and a transverse dual echo fast field echo sequence under a 20 s expiration breath hold. The Analyze9^™^ software (Mayo Foundation, Rochester, MN, USA) was used to manually segment each regional colon volume from the coronal image slices. Each colon region was identified and a 3D representation of the morphology, from which the volume of each regional colon was measured, was built (Fig. 1). Lastly, motility scans of the ascending colon (AC) involved a single sagittal slice positioned centrally through the AC, using a balanced turbo field echo sequence (TR/TE = 3/1.5 ms, FA = 70°, FOV = 330 × 228 × 15 mm^3^, ACQ res = 1.5 × 1.5 × 15 mm^3^), which was scanned repetitively every second for 2 min (cine MRI) during which time the participants were allowed to breathe gently. The data were registered using the dual registration of abdominal motion methodology.^9^ The registration process first removes breathing effects from the images and then parameterizes the motion of the tissue within the images over time to allow lines and regions of interest to be automatically tracked through the time series using custom written software in Matlab^®^ (The MathsWorks Inc, Natick, MA, USA). Following registration, an observer drew a series of lines across the AC on the median image, perpendicular to the main axis of the AC and with the edges of the lines touching the colon walls.^17^ These lines were then automatically propagated through the time series using the information from image registration. Any changes in line with length indicated movement of the colonic wall (i.e., a contraction or expansion of the lumen). A motility index was defined as the fraction of time points, summed across all lines drawn (line length smoothed to reduce noise) which had a rate of change in line length (i.e., wall velocity) between consecutive time points of more than 0.5 mm/s. Hereafter this is referred to as line analysis _0.5mm/s_ index.^17^ This index had been validated against observer measurements of wall motion^11^ and has an excellent correlation with observer scoring across a wide range of colonic motility.^17^ Sensitivity to distension was assessed by dividing bloating score at the time of peak distension (120 min from start of Moviprep^®^ ingestion) by the simultaneously recorded total colonic volumes.

### Repeatability, inter‐observer variability, and normal ranges

All our images were analyzed by an operator blind to the patient details to avoid bias. Using our standard techniques the inter‐observer variability for colonic volumes is 5% (Pritchard SE *et al*. unpublished data).^8^ For colonic transit, the inter‐observer variability is also good with previously reported intra‐class correlation coefficient = 0.78; while in previous studies the day‐to‐day repeatability of transit was acceptable with intra‐class correlation coefficient of 0.61.^10^ For the line analysis _0.5mm/s_ index of motility intra‐ and inter‐observer agreement was excellent, with previously reported intra‐class correlation coefficients of 0.96 and 0.95, respectively.^17^

We have established that the median WAPS in a group of healthy volunteers was 0.8 (IQR 0–1.6) based on our previous HV study.^10^ We defined the upper limit of normal (ULN) for the WAPS as exceeding the 95th centile above normal value, that is, more than 2.2. The normal colonic volumes based on the previous study^8^ were 203 ± 75 mL for AC volume, 198 ± 79 mL for transverse colon volume, and 60 ± 86 mL for descending colon volumes giving upper limit of normal of 343, 325, and 282 mL, respectively. The normal SBWC based on our previous study^15^ was 81 (37–130) mL giving the upper limit normal of 127 mL. The upper limit of normal for time to first bowel movement in a cohort of healthy volunteers from our previous study^11^ was 190 min. The upper limit of normal for sensitivity to distension at 2 h post Moviprep^®^ in a cohort of healthy volunteers from our previous study^11^ was 5.2/L.

### Power and statistical analysis

All statistical analyses were carried out using the GraphPad Prism version 6.0 for Windows (GraphPad Software, La Jolla, CA, USA). D'Agostino and Pearson omnibus normality test was used to assess distribution of data. Normally distributed data are expressed as mean ± SD and non‐normally distributed data are expressed as median (interquartile range; IQR). Normally distributed data were analyzed using the unpaired *t*‐test, one‐way anova and two‐way anova while non‐normally distributed data were analyzed using Mann–Whitney test and Kruskal–Wallis test.

#### Sample size

Our previous study of the effect of another non‐absorbable osmotic laxative mannitol^15^ gave a mean (SD) change in SBWC at 40 min postprandially after ingesting 300 mL glucose of 6 (39.5) mL. Using *n* = 12, we calculated we could detect an increase in 55 mL with 90% power which was very much less than predicted from theoretical considerations which suggest a change in >1000 mL. We planned to use 24 per group to ensure we could assess our secondary endpoints for which there are no data with which to perform a power calculation. There was no previous study using MRI to assess small and large bowel motility/function in IBS‐C and FC so we were not able to perform a power calculation for these parameters.

## Results

Forty‐eight participants were recruited into the study (for Consort diagram see Fig. S1); however, five did not complete the study, one due to diarrhea the day before, three due to withdrawal of consent, and one became pregnant. Thus, 23 FC and 20 IBS‐C were included in the analysis.

### Demographics

Participants were expected to be mostly middle‐aged females with mild anxiety and somatization. There were no differences in the baseline demographics between the FC and IBS‐C group (Table 1). The usage of laxative(s) prior to recruitment to the study was similar in the two groups of patients who used one to three laxatives, mostly osmotic and stimulatory agents with 8 and 11, respectively, having tried and failed the newer prokinetic prucalopride. While baseline abdominal pain in the week preceding the study day tended to be higher in IBS, this was not statistically significant. Other baseline symptoms for both groups, particularly stool frequency and consistency were similar, the only exception being the significantly higher urgency score for the IBS‐C patients compared to the FC patients (Table 2).

**Table 1:** Baseline characteristics for FC and IBS‐C patients in the week preceding the MRI study day

Mean (SD)	FC (*n* = 23)	IBS‐C (*n* = 20)	*p*‐value
Age	47 (35–51)	39 (27–53)	0.18
Male : female	2 : 21	0 : 20	
Anxiety score (range 0–21)	8.5 (5.4)	7.8 (5.4)	0.68
Depression score (range 0–21)	4.5 (3–12)	4.0 (2.0–6.8)	0.20
PHQ12SS	6.6 (3.9)	7.1 (4.5)	0.74

Values are mean (SD) if normally distributed data and median (interquartile range) if non‐normally distributed data. FC, functional constipation; IBS‐C, irritable bowel syndrome with constipation.

**Table 2:** Baseline abdominal symptom scores during week preceding the MRI study day

	FC	IBS‐C	*p*‐value
Abdominal pain (0–10)	0.71 (0.29–2.29)	1.57 (0.96–1.86)	0.29
Urgency (0–10)	0.14 (0–0.71)	0.32 (0–0.64)	0.04
Bloating (0–10)	1.39 (0.91)	1.69 (0.79)	0.28
Average stool frequency/week	0.57 (0.29–0.71)	0.64 (0.54–1.0)	0.15
Average stool consistency (1–7)	2.66 (1.68)	2.57 (1.42)	0.85

Values are mean (SD) if normally distributed data and median (interquartile range) for non‐normally distributed data. FC, functional constipation; IBS‐C, irritable bowel syndrome with constipation.

### MRI parameters

#### Gut transit

Weighted average position score exceeded the ULN in 82% of FC and 47% of IBS‐C patients. The median WAPS was 3.6 (2.5–4.2) for FC, significantly greater than the 2.0 (1.5–3.2) for IBS‐C indicating slower transit, *p* = 0.01. When compared with the healthy volunteers in our previous study,^10^ both patient groups showed significantly slower transit, Kruskal–Wallis test *p* < 0.01.

#### Intestinal volumes

**Figure 2:**
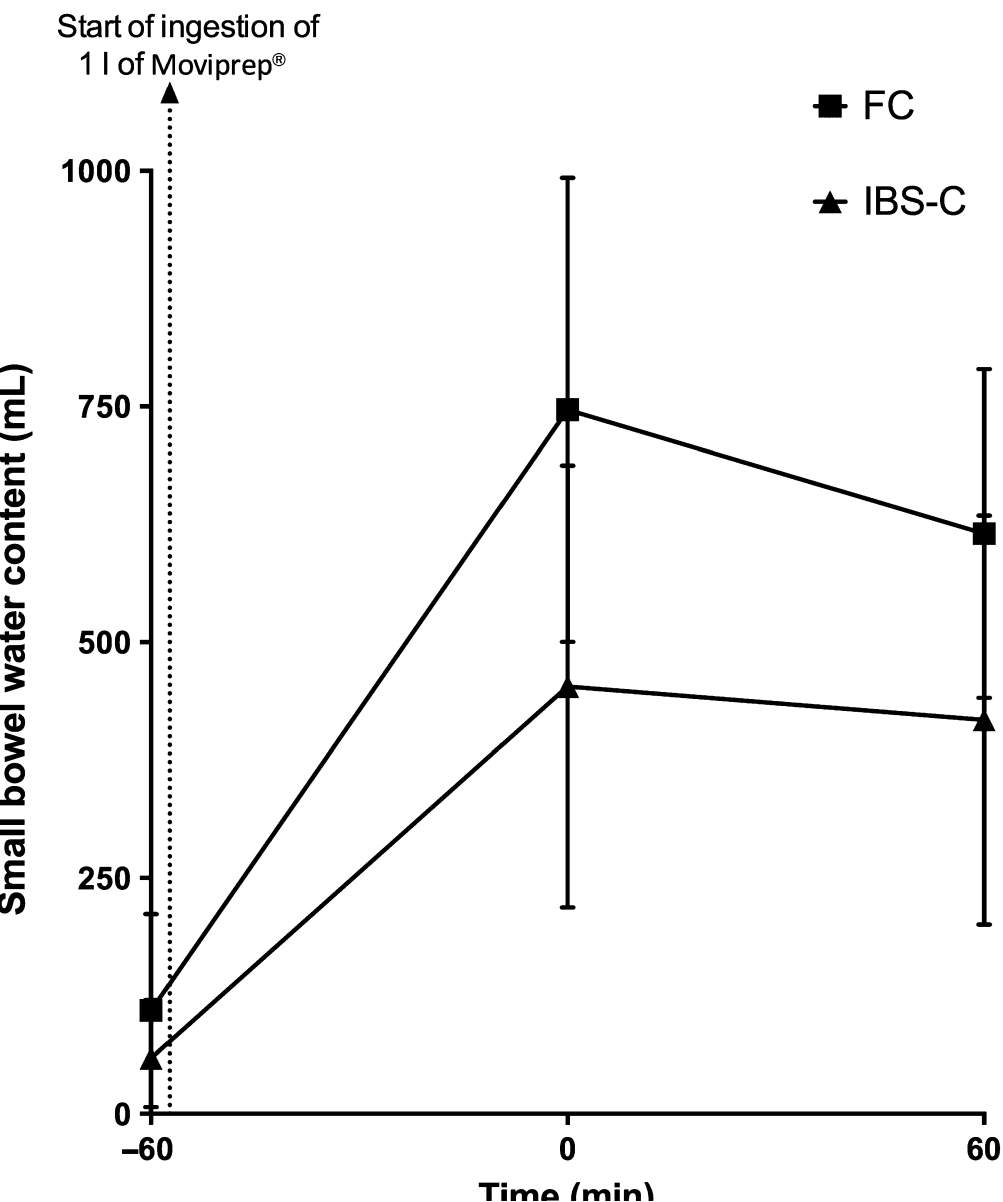
Small bowel water content (SBWC) in the functional constipation (FC) and irritable bowel syndrome with constipation (IBS‐C) patient groups during the initial 2 h of the study. The baseline scan is at time −60 min and the arrow shows the start of Moviprep^®^ ingestion. Time 0 is time of completion of Moviprep^®^ ingestion. Small bowel water content rose significantly over time for FC. FC patients had significant elevated fasting SBWC at baseline (time −60 min); *p* < 0.01, Time 0 after completion of Moviprep^®^; *p* < 0.01 and 1 h after completion of Moviprep^®^; *p* = 0.03, compared to IBS‐C.

Small bowel: Fasting SBWC was above the upper limit of normal for 30% of FC and 15% IBS‐C, the value for FC being significantly higher at 83 (63–142) mL compared to IBS‐C 39 (15–70) mL, respectively, *p* < 0.01 (Fig. S2). Small bowel water content for both groups peaked at 1 h following the start of ingestion of Moviprep^®^ before declining (Fig. 2). At each time point including the fasting baseline, SBWC was significantly higher in FC compared to IBS‐C (Fig. 2). Further SBWC measurements were not considered as patients had water *ad libitum* after 2 h following ingestion of Moviprep^®^.

**Figure 3:**
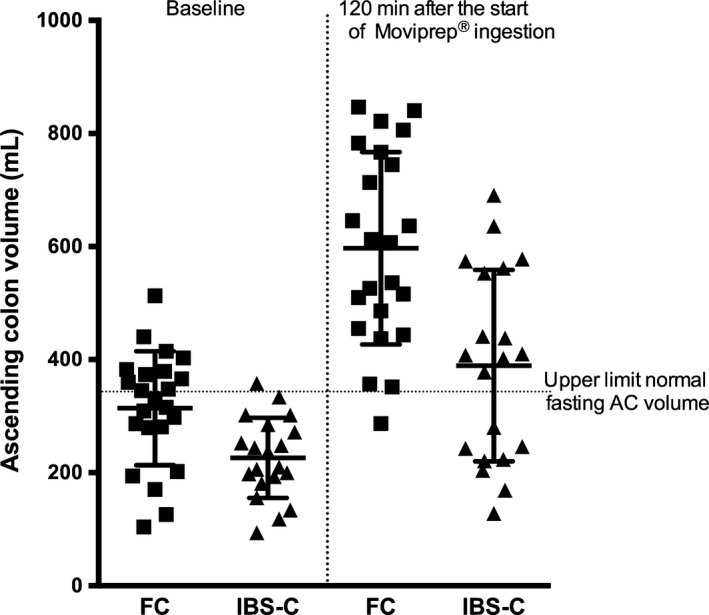
Ascending colon volumes in functional constipation (FC) and irritable bowel syndrome with constipation (IBS‐C) patients were 314 (101) and 597 (170) mL for FC, and 226 (71) mL and 389 (169) mL for IBS‐C at baseline and 120 min after the start of Moviprep^®^ ingestion, respectively. Both fasting and 120‐min values for FC were significantly greater than IBS‐C, both *p* < 0.01.

Large bowel: Forty‐eight percent of FC and 5% of IBS‐C had baseline AC volumes above the upper limit of normal. These were significantly higher in the FC group compared to IBS‐C group (Table 3 and Fig. 3). One h following completion of Moviprep^®^, the AC volumes were significantly increased in the FC group compared to IBS‐C (Table 3 and Fig. 3).

**Table 3:** Colonic volumes, motility of ascending colon, and sensitivity index between FC and IBS‐C groups

	FC	IBS‐C	*p*‐value
Baseline ascending colonic volumes (mL)	314 (101)	226 (71)	<0.01
Ascending colon volumes at 120 min	597 (170)	389 (169)	<0.01
Baseline total colonic volumes (mL)	847 (280)	662 (240)	0.03
Total colon volumes at 120 min	1505 (387)	1039 (418)	<0.01
Motility of ascending colon at 120 min (line analysis_0.5mm/s_ index)	0.055 (0.044)	0.107 (0.070)	<0.01

Values are mean (SD). FC, functional constipation; IBS‐C, irritable bowel syndrome with constipation.

**Figure 4:**
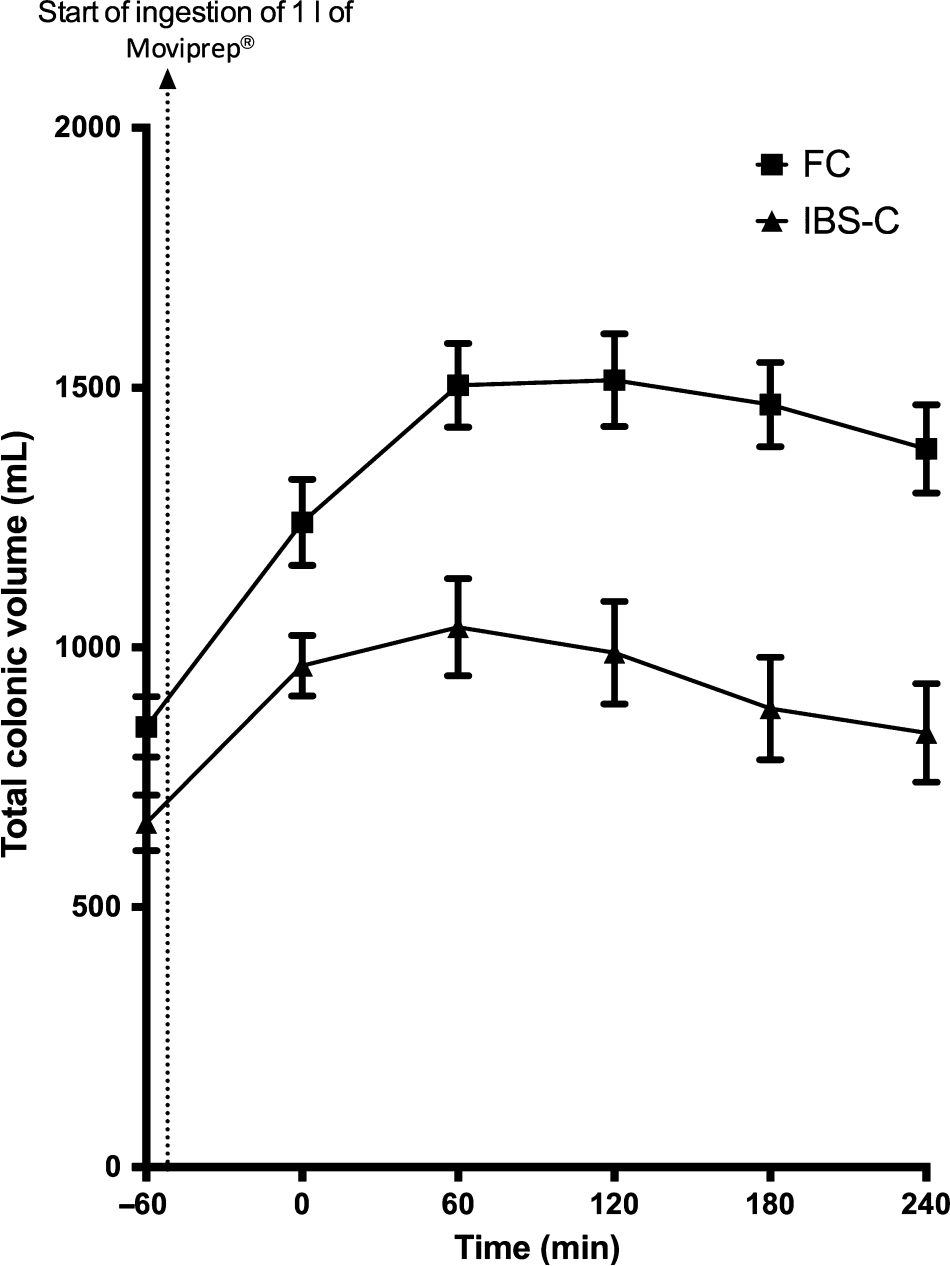
Total colonic volumes over time for functional constipation (FC) and irritable bowel syndrome with constipation (IBS‐C) patients. The time is from completion of Moviprep^®^ ingestion. As can be seen, total colon volumes peaked at 60 min. Two‐way anova showed a significant difference both over time (*p* < 0.01) and between groups (*p* < 0.01), with significantly higher values for FC. The interaction effect was significant at *p* < 0.01.

When the total colonic volume was measured, FC had significantly higher total colonic volume compared to IBS‐C (Table 3). As can be seen in Fig. 4, the total colonic volume for FC nearly doubled from baseline at 1 h following completion of ingestion of 1 L Moviprep^®^, and remained significantly higher during the subsequent 3 h when compared to IBS‐C. There was a significant effect of patient group (*p* < 0.01) and of time (*p* < 0.01), with a significant interaction between the two (*p* < 0.01) as shown by two‐way anova.

#### Motility index

**Figure 5:**
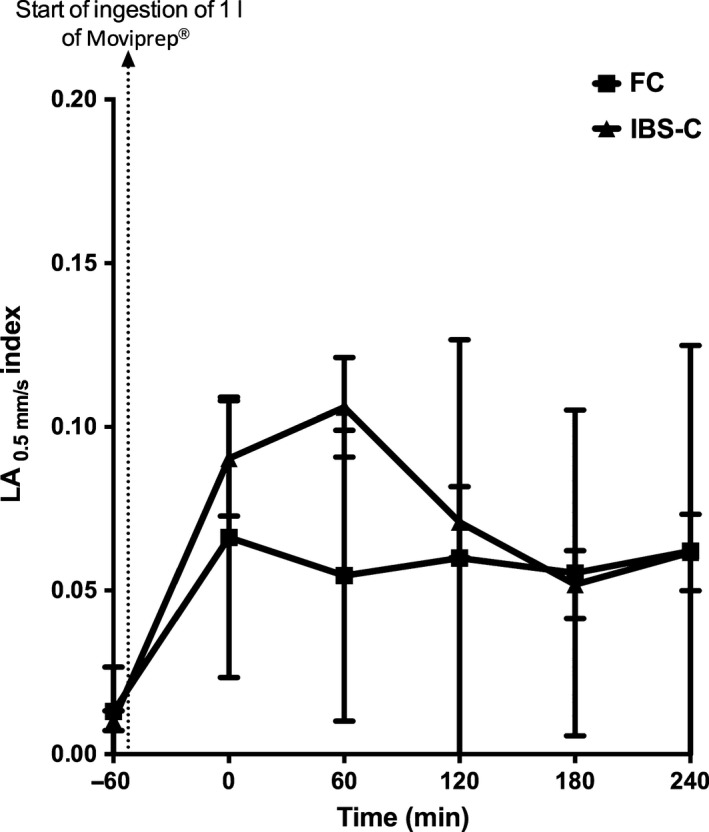
Motility of the ascending colon for functional constipation (FC) and irritable bowel syndrome with constipation (IBS‐C) patients during the study day. Motility for IBS‐C rapidly rose from baseline and peaked at 120 min from the start of Moviprep^®^ ingestion when the motility for IBS‐C was significantly elevated at 0.107 (0.070) compared to FC 0.055 (0.044), *p* < 0.01.

The motility of the AC based on the line analysis _0.5mm/s_ index rose rapidly after Moviprep ingestion, but was significantly lower in FC compared to IBS‐C at 120 min from the start of Moviprep^®^ ingestion (Table 3 and Fig. 5).

#### Bowel habit following stimulation with laxative

All FC patients had fewer bowel movements in the 24‐h period following ingestion of Moviprep^®^ than our lower limit of normal which is 6. Functional constipation patients had only three (2–5) bowel movements, significantly less than the IBS‐C patients who had seven (6–10) bowel movements/24 h, *p* < 0.01. The time to the first bowel movement following ingestion of Moviprep^®^ was above our upper limit of normal in 60% of FC patients but only 15% of the IBS‐C patients. Mean value was significantly longer in FC group compared with IBS‐C, being 295 (116–526) min and 84 (49–111) min in FC and IBS‐C, respectively, *p* < 0.01 (Fig. S3).

### Correlation between time to first bowel movement after provocation with laxative and MRI parameters

Those with distended ACs following Moviprep^®^ took longer to open their bowel. Time to first bowel movement correlated positively with AC volume at 2 h post Moviprep^®^, Spearman *r* = 0.44, *p* < 0.01. It also correlated positively with the fasting SBWC, Spearman *r* = 0.34, *p* = 0.04.

### Predictors of symptoms

Symptoms in general correlated poorly with objective MRI parameters. Bloating was not linked to objective distension (based on the 10‐cm VAS‐bloating score during the study period) moreover sensitivity to distension at 120 min post Moviprep^®^, showed no difference between the groups being 4.84 (2.64) and 5.62 (4.40) per L, *p* = 0.49 for FC and IBS‐C, respectively. The sensitivity to distension at 120 min post Moviprep^®^ was above our upper limit of normal in 55% of IBS‐C patients and 35% of FC patients.

## Discussion

This is the first report of an objective, dynamic assessment of colonic function, performed non‐invasively using MRI in chronic constipated patients and as such provides much new data. Very early studies using X‐ray images had provided anecdotal details of colonic motility and response to eating.^18^ However, the realization of the dangers of irradiation brought such studies to an abrupt end. Subsequent studies have used transit of radio‐opaque markers^13^ and clearance of isotope from the colon^19^ as surrogate markers of motility. These correlate reasonably well with symptoms, but of course provide little detail of colonic wall movements.^20^

Direct measurement of colonic motility using manometry has been used in specialist centers but is technically demanding, requiring bowel preparation, and colonoscopy to position the tube, which is unpleasant and carries a small but definite risk for the patient. Furthermore, given substantial diurnal variation in colonic motility adequate assessment requires prolonged (up to 24 h) recording which imposes a further burden on the patient.^5^ This has limited its use despite the exquisite detail it provides.^21^

Our technique is by contrast extremely easy to administer and very acceptable to patients. Furthermore, our test is directly related to the symptoms patients complain of, namely unresponsiveness to laxatives, and gives a clear indication of the mechanisms underlying constipation.

By providing a large distending stimulus we have been able to show distinct motor responses which are impaired in most patients with FC. Most IBS‐C patients in contrast show a normal initial response but in the latter half of the study this appears to tail off more rapidly than we expected from our previously published studies of healthy volunteers.^11^ Motility was assessed using a validated, automatic technique that reduces the time to obtain a report and improves inter‐observer variability.^17^ It will also make it feasible to analyze longer time periods, though by using a provocation test with a strong stimulus such as Moviprep^®^, less time is required than if waiting for spontaneous contractions, which are often erratic. Whether these responses are useful clinically to predict response to therapy requires further study but anecdotally it is our impression that those with a hypomotile colon seem to respond well to prokinetics such as prucalopride while those with normal or hyperactive colons tend to get pain and diarrhea.

The observation of colonic volumes in functional bowel disorders is novel and its full significance still unclear. Using the MRI to visualize the physiology of the undisturbed large bowel, we had found that the fasting AC of patients with FC is significantly larger than those with IBS‐C. When compared with our previous study, the AC volume in FC [314 (100) mL] is significantly larger than the healthy controls [202.9 (75.0) mL] and IBS with diarrhea [median 188 (165–251) mL], Kruskal–Wallis, *p* < 0.01.^8^ In contrast there was no difference in the AC volume in IBS‐C when comparing to similar healthy control, *p* = 0.21. Whether treatment will alter resting volumes requires further study.

Constipation is associated with slow transit^22^ and diarrhea with fast whole gut transit^23^ and accelerated clearance of the AC,^24^ but in each case the overlap with normal is substantial, as is the day to day variability at around 25%.^25^ This is undoubtedly because transit depends on many uncontrolled factors such as diet, microbiota, psychological factors, and motility.

A dynamic function test using a large stimulus in a controlled setting allows us to overcome some of this background noise and in patients with FC, show a clear difference from both normal and IBS‐C. This is important because with the exception of pain and urgency our FC and IBS‐C patients had very similar clinical features including bloating, infrequent bowel movements, hard stools, and pain. Despite this they have very different transit and as we show, differing underlying pathophysiology.

The small bowel has been largely ignored in studies of constipation in the past because there was no easy way of assessing its function. Magnetic resonance imaging provides a new way of addressing its role. We found the fasting SBWC to be significantly larger in FC compared to IBS‐C suggesting a pan‐intestinal defect. In our previous study by Marciani *et al*.,^15^ we found that patients with diarrhea due to IBS have reduced fasting SBWC, which may reflect increased tone and faster orocecal transit time. This may imply that the larger fasting SBWC seen in our FC group reflects reduced small bowel tone and prolonged orocecal transit time, but we did not measure this as our previous study suggested MRI assessment of OCTT is less reliable.^10^

We tried to assess visceral hypersensitivity non‐invasively by looking at symptoms particularly bloating following the distension of the AC by Moviprep^®^. Overall visceral hypersensitivity between the patients (both FC and IBS‐C) was significantly higher compared to healthy volunteers giving a mean (SD) of 5.2 (3.5) for the patients and 2.0 (1.7) for healthy volunteers (*p* < 0.01). Unfortunately there is substantial overlapping between the patient groups, possibly because the FC group starts from a larger initial volume, which may make the arrival of large volumes of fluid more painful than in IBS patients, who start with a relatively normal AC.

### Limitations

An important limitation in our study was the fact that the WAPS was previously validated in healthy controls that had a median (IQR) of 0.8 (0–1.6).^10^ Extrapolating beyond this range may not be valid and a future validation study in a cohort of constipated patients will be required. We did not standardize patients' diet/fiber intake prior to the study but in the future this might reduce the variability in fasting values.

### Clinical implications

Our test requires only a standard MRI scanner which is available in most centers. Its cost compares favorably with the current alternative way of assessing disordered colonic motility namely manometry. It is also comparable to the cost of colonoscopy, a test widely performed in evaluating constipation but which yields no information about colonic function and is rarely of value. While currently only available in specialist centers, its use in very severe cases in whom colectomy or sacral nerve stimulator implantation is contemplated could be easily justified if it prevents an IBS‐C patient from undergoing unnecessary and ineffective treatments.

Even for those without access to MRI for such patients, the Moviprep^®^ challenge could be used without imaging, as defecation after ingestion of Moviprep^®^ within 230 min identifies 95% of IBS‐C while only being found in 45% of FC. This is useful because it should prevent the use of strong stimulant laxatives in IBS‐C, as this can cause further abdominal pain and suggest that an agent with both laxative and pain‐relieving properties such as Linaclotide or Lubiprostone might be a better treatment for this group of patients.^26^, ^27^

Even though CC is common, diagnosis of this condition is mainly based on patient‐reported symptoms. This can be unreliable as very often there is a lack of agreement between physician and patients when defining constipation.^3^, ^28^ In this study, we were able to objectively measure intestinal volumes and colonic motility in patients with chronic constipation. It is possible that in the future patients could be categorized by these objective parameters rather than the current symptom‐based classification. This may allow a better prediction of response to specific treatments.

We have shown that this colonic function test is patient acceptable, technically undemanding and shows important differences in colonic physiology between patients with very similar symptoms. Its use could contribute to individualizing patient care in this common but poorly treated condition. Finally, being non‐invasive and involving no ionizing radiation this test is eminently suitable for evaluating new treatments for disordered intestinal motility.

## Funding

This is a summary of independent research funded by the National Institute for Health Research Biomedical Research Unit and the MRC. The views expressed are those of the author(s) and not necessarily those of the NHS, the NIHR, the MRC or the Department of Health.

## Disclosure

Ching Lam: None. Gemma Chaddock: None. Luca Marciani: None. Carolyn Costigan: None. Joe Paul: None. Eleanor Cox: None. Caroline Hoad: None. Alex Menys: Director and Shareholder of Motilent Ltd. Susan Pritchard: None. Klara Garsed: None. Stuart Taylor: None. David Atkinson: None. Penny Gowland: None. Robin Spiller has received research funding from Lesaffre and Ironwood. He has also acted on Advisory Boards for Almirall, Yuhan Corporation, Ibsen and Danone and received speaker's fees from Menarini.

## Author Contribution

CL was involved in recruitment of participants into study, study concept and design, analysis and interpretation of data, statistical analysis, administrative and material support, study supervision; GC was involved in study supervision, acquisition and analysis of data; LM contributed to study supervision, study concept and design, statistical analysis and technical support; CC provided technical support; JP performed analysis of data; EC provided technical support; CH was involved in study concept and design, technical support, analysis of data; AM performed image registration and provided technical support; SP was involved in technical support and analysis of data; KG was involved in study concept and design and administrative support; ST was involved in supervision for image registration; DA was involved in supervision for motility assessment; PG obtained funding, and was involved in study concept and design, interpretation of data; RS obtained funding, and was involved in study concept and design, interpretation of data, study supervision, recruitment. All authors assisted in drafting of the manuscript.

## Supporting information


**Figure S1** Consort diagram showing recruitment.
**Figure S2** Fasting small bowel water content (SBWC) in the functional constipation (FC) and irritable bowel syndrome with constipation (IBS‐C) patient groups as measured using MRI.
**Figure S3** Time to first bowel movement (min) following ingestion of Moviprep^®^ for functional constipation (FC) and irritable bowel syndrome with constipation (IBS‐C) patients.

## References

[1] Higgins PD , Johanson JF . Epidemiology of constipation in North America: a systematic review. Am J Gastroenterol 2004; 99: 750–9. 15089911 10.1111/j.1572-0241.2004.04114.x

[2] Johanson JF , Kralstein J . Chronic constipation: a survey of the patient perspective. Aliment Pharmacol Ther 2007; 25: 599–608. 17305761 10.1111/j.1365-2036.2006.03238.x

[3] Longstreth GF , Thompson WG , Chey WD , Houghton LA , Mearin F , Spiller RC . Functional bowel disorders. Gastroenterology 2006; 130: 1480–91. 16678561 10.1053/j.gastro.2005.11.061

[4] Wong RK , Palsson OS , Turner MJ , Levy RL , Feld AD , von Korff M , Whitehead WE . Inability of the Rome III criteria to distinguish functional constipation from constipation‐subtype irritable bowel syndrome. Am J Gastroenterol 2010; 105: 2228–34. 20502449 10.1038/ajg.2010.200 PMC3786710

[5] Rao SS , Sadeghi P , Beaty J , Kavlock R . Ambulatory 24‐hour colonic manometry in slow‐transit constipation. Am J Gastroenterol 2004; 99: 2405–16. 15571589 10.1111/j.1572-0241.2004.40453.x

[6] Hasler WL , Saad RJ , Rao SS , Wilding GE , Parkman HP , Koch KL , McCallum RW , Kuo B *et al* Heightened colon motor activity measured by a wireless capsule in patients with constipation: relation to colon transit and IBS. Am J Physiol Gastrointest Liver Physiol 2009; 297: G1107–14. 19808653 10.1152/ajpgi.00136.2009

[7] Hoad CL , Marciani L , Foley S , Totman JJ , Wright J , Bush D , Cox EF , Campbell E *et al* Non‐invasive quantification of small bowel water content by MRI: a validation study. Phys Med Biol 2007; 52: 6909–22. 18029983 10.1088/0031-9155/52/23/009

[8] Pritchard SE , Marciani L , Garsed KC , Hoad CL , Thongborisute W , Roberts E , Gowland PA , Spiller RC . Fasting and postprandial volumes of the undisturbed colon: normal values and changes in diarrhea‐predominant irritable bowel syndrome measured using serial MRI. Neurogastroenterol Motil 2014; 26: 124–30. 24131490 10.1111/nmo.12243 PMC3995006

[9] Menys A , Hamy V , Makanyanga J , Hoad C , Gowland P , Odille F , Taylor SA , Atkinson D . Dual registration of abdominal motion for motility assessment in free‐breathing data sets acquired using dynamic MRI. Phys Med Biol 2014; 59: 4603–19. 25079109 10.1088/0031-9155/59/16/4603

[10] Chaddock G , Lam C , Hoad CL , Costigan C , Cox EF , Placidi E , Thexton I , Wright J *et al* Novel MRI tests of orocecal transit time and whole gut transit time: studies in normal subjects. Neurogastroenterol Motil 2014; 26: 205–14. 24165044 10.1111/nmo.12249 PMC4285997

[11] Marciani L , Garsed KC , Hoad CL , Fields A , Fordham I , Pritchard SE , Placidi E , Murray K *et al* Stimulation of colonic motility by oral PEG electrolyte bowel preparation assessed by MRI: comparison of split vs single dose. Neurogastroenterol Motil 2014; 26: 1426–36. 25060551 10.1111/nmo.12403 PMC4321061

[12] Cremonini F , Mullan BP , Camilleri M , Burton DD , Rank MR . Performance characteristics of scintigraphic transit measurements for studies of experimental therapies. Aliment Pharmacol Ther 2002; 16: 1781–90. 12269971 10.1046/j.1365-2036.2002.01344.x

[13] Metcalf AM , Phillips SF , Zinsmeister AR , MacCarty RL , Beart RW , Wolff BG . Simplified assessment of segmental colonic transit. Gastroenterology 1987; 92: 40–7. 3023168 10.1016/0016-5085(87)90837-7

[14] O'Donnell LJ , Virjee J , Heaton KW . Detection of pseudodiarrhoea by simple clinical assessment of intestinal transit rate. BMJ 1990; 300: 439–40. 2107897 10.1136/bmj.300.6722.439 PMC1662249

[15] Marciani L , Cox EF , Hoad CL , Pritchard S , Totman JJ , Foley S , Mistry A , Evans S *et al* Postprandial changes in small bowel water content in healthy subjects and patients with irritable bowel syndrome. Gastroenterology 2010; 138: 469–77, 77 e1. 19909743 10.1053/j.gastro.2009.10.055

[16] Marciani L , Wright J , Foley S , Hoad CL , Totman JJ , Bush D , Hartley C , Armstrong A *et al* Effects of a 5‐HT(3) antagonist, ondansetron, on fasting and postprandial small bowel water content assessed by magnetic resonance imaging. Aliment Pharmacol Ther 2010; 32: 655–63. 20626735 10.1111/j.1365-2036.2010.04395.x

[17] Hoad CL , Menys A , Garsed K , Marciani L , Hamy V , Murray K , Costigan C , Atkinson D *et al* Colon wall motility: comparison of novel quantitative semi‐automatic measurements using cine MRI. Neurogastroenterol Motil. DOI: 10.1111/nmo.12727. 10.1111/nmo.12727 26612075

[18] Hurst AF . Constipation and Allied Intestinal Disorders. London: Forgotten Books, 2013 (Original work published 1919).

[19] Camilleri M , Hasler WL , Parkman HP , Quigley EM , Soffer E . Measurement of gastrointestinal motility in the GI laboratory. Gastroenterology 1998; 115: 747–62. 9721173 10.1016/s0016-5085(98)70155-6

[20] Deiteren A , Camilleri M , Bharucha AE , Burton D , McKinzie S , Rao AS , Zinsmeister AR . Performance characteristics of scintigraphic colon transit measurement in health and irritable bowel syndrome and relationship to bowel functions. Neurogastroenterol Motil 2010; 22: 415–23, e95. 20025675 10.1111/j.1365-2982.2009.01441.x PMC2852474

[21] Dinning PG , Szczesniak MM , Cook IJ . Twenty‐four hour spatiotemporal mapping of colonic propagating sequences provides pathophysiological insight into constipation. Neurogastroenterol Motil 2008; 20: 1017–21. 18513217 10.1111/j.1365-2982.2008.01147.x

[22] Rao SS , Kuo B , McCallum RW , Chey WD , DiBaise JK , Hasler WL , Koch KL , Lackner JM *et al* Investigation of colonic and whole‐gut transit with wireless motility capsule and radiopaque markers in constipation. Clin Gastroenterol Hepatol 2009; 7: 537–44. 19418602 10.1016/j.cgh.2009.01.017

[23] Cann PA , Read NW , Brown C , Hobson N , Holdsworth CD . Irritable bowel syndrome: relationship of disorders in the transit of a single solid meal to symptom patterns. Gut 1983; 24: 405–11. 6840614 10.1136/gut.24.5.405 PMC1419989

[24] Vassallo M , Camilleri M , Phillips SF , Brown ML , Chapman NJ , Thomforde GM . Transit through the proximal colon influences stool weight in the irritable bowel syndrome. Gastroenterology 1992; 102: 102–8. 1727743 10.1016/0016-5085(92)91789-7

[25] Degen LP , Phillips SF . Variability of gastrointestinal transit in healthy women and men. Gut 1996; 39: 299–305. 8977347 10.1136/gut.39.2.299 PMC1383315

[26] Chey WD , Lembo AJ , Lavins BJ , Shiff SJ , Kurtz CB , Currie MG , MacDougall JE , Jia XD *et al* Linaclotide for irritable bowel syndrome with constipation: a 26‐week, randomized, double‐blind, placebo‐controlled trial to evaluate efficacy and safety. Am J Gastroenterol 2012; 107: 1702–12. 22986437 10.1038/ajg.2012.254

[27] Rao S , Lembo AJ , Shiff SJ , Lavins BJ , Currie MG , Jia XD , Shi K , MacDougall JE *et al* A 12‐week, randomized, controlled trial with a 4‐week randomized withdrawal period to evaluate the efficacy and safety of linaclotide in irritable bowel syndrome with constipation. Am J Gastroenterol 2012; 107: 1714–24; quiz p. 25. 22986440 10.1038/ajg.2012.255 PMC3504311

[28] Probert CS , Emmett PM , Cripps HA , Heaton KW . Evidence for the ambiguity of the term constipation: the role of irritable bowel syndrome. Gut 1994; 35: 1455–8. 7959204 10.1136/gut.35.10.1455 PMC1375024

