# Supplementary material for: Colonic response to laxative ingestion as assessed by MRI differs in constipated irritable bowel syndrome compared to functional constipation
Source: Neurogastroenterol Motil. 2016 Feb 12;28(6):861–70. doi: 10.1111/nmo.12784 (PMC4949702; doi:10.1111/nmo.12784)
Supplement: Supplementary file 1 — Figure S1 Consort diagram showing recruitment. Figure S2 Fasting small bowel water content (SBWC) in the functional constipation (FC) and irritable bowel syndrome with constipation (IBS‐C) patient groups as measured using MRI. Figure S3 Time to first bowel movement (min) following ingestion of Moviprep® for functional constipation (FC) and irritable bowel syndrome with constipation (IBS‐C) patients. [file NMO-28-861-s001.docx]

**Supporting information:**

**
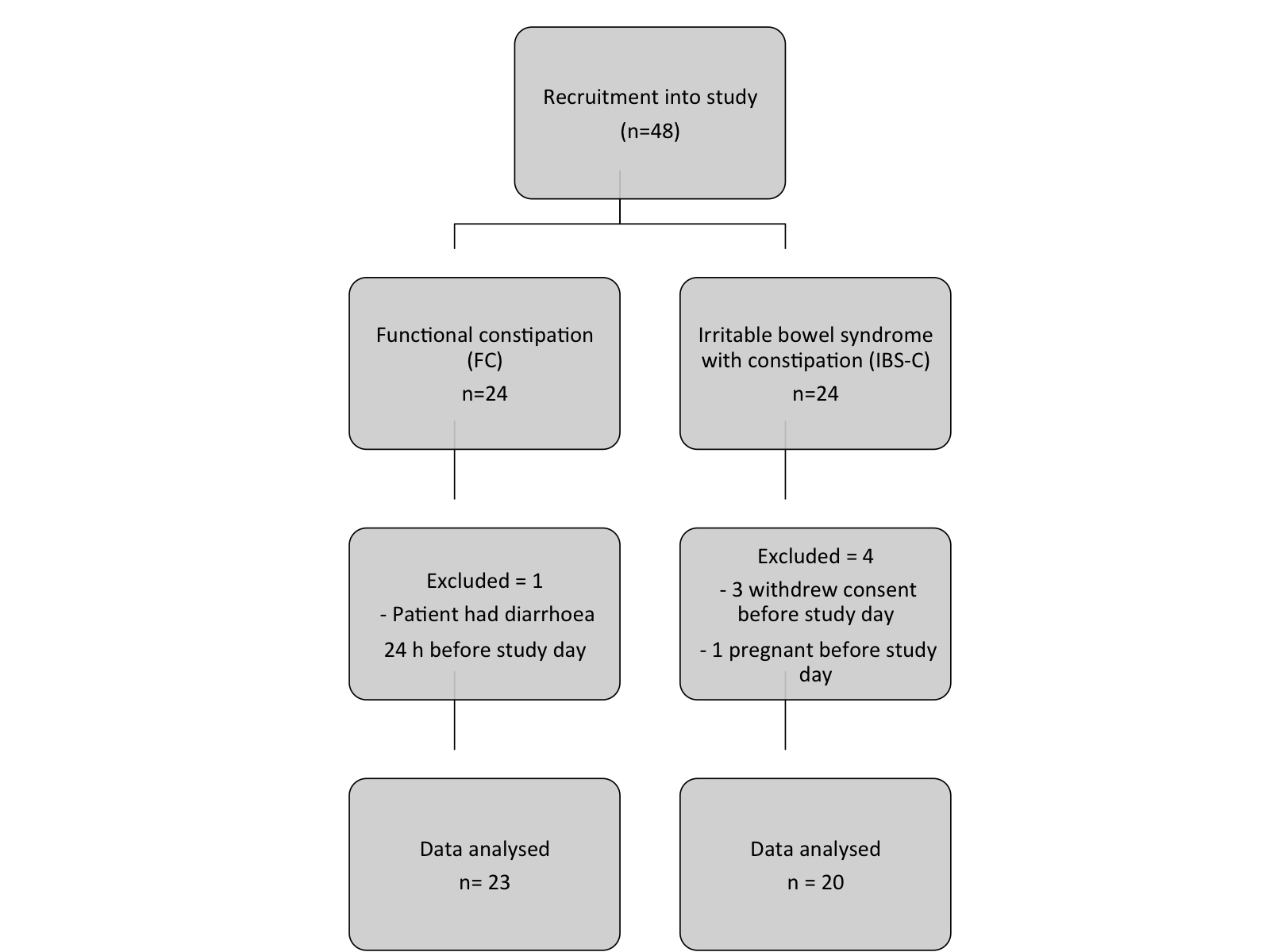
**

**Supplemental Figure 1: Consort diagram showing recruitment.**

**
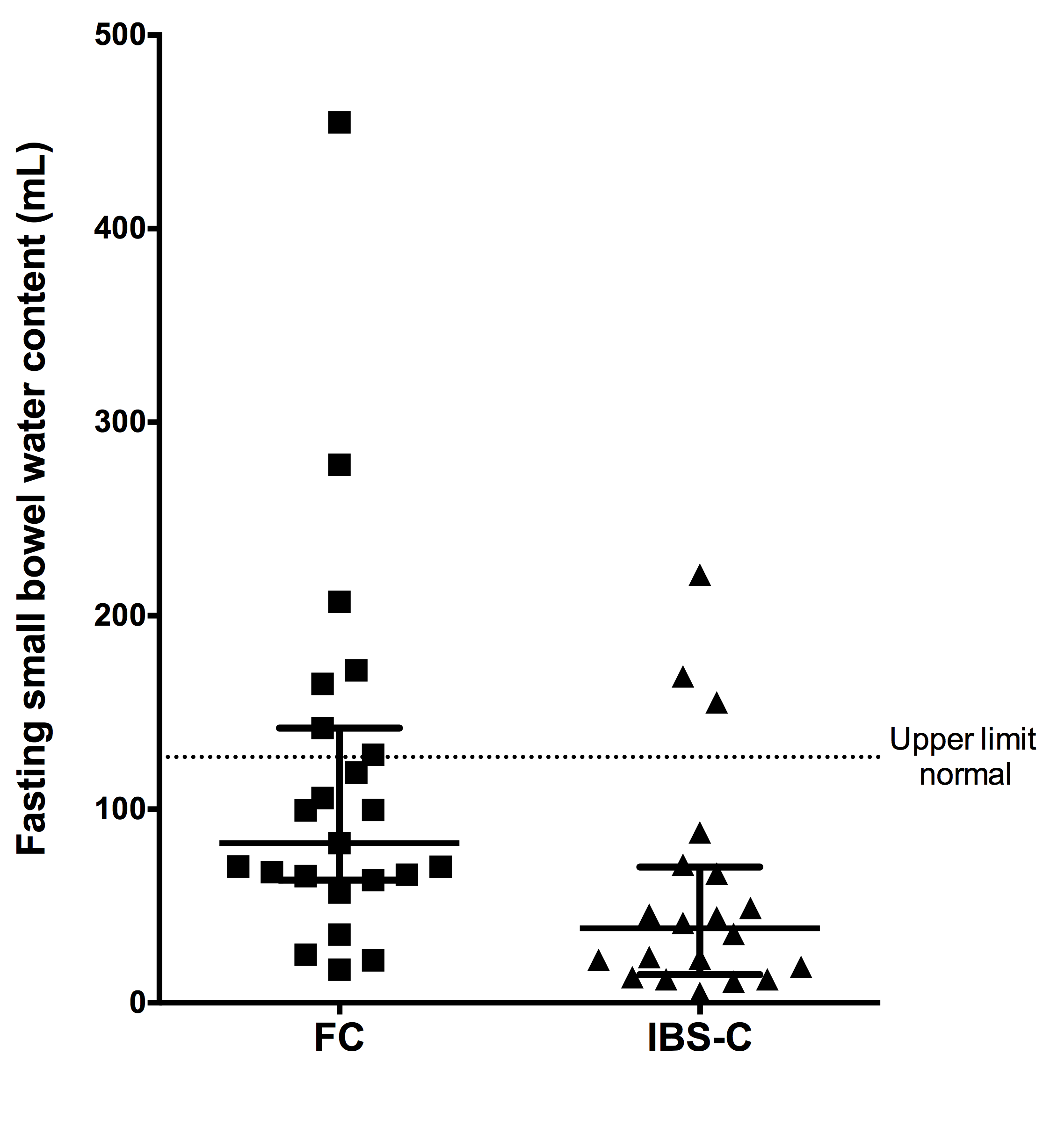
**

**Supplemental Figure 2: Fasting small bowel water content (SBWC) in the Functional Constipation (FC) and Irritable bowel syndrome with constipation (IBS-C) patient groups as measured using MRI. Patients meeting FC criteria had significantly greater faster small bowel water content, p<0.01. Using our upper limit of normal from previous studies 7 out of 23 (30%) of FC patients were elevated but only 3 out of 20 (15%) of IBS-C.**

**
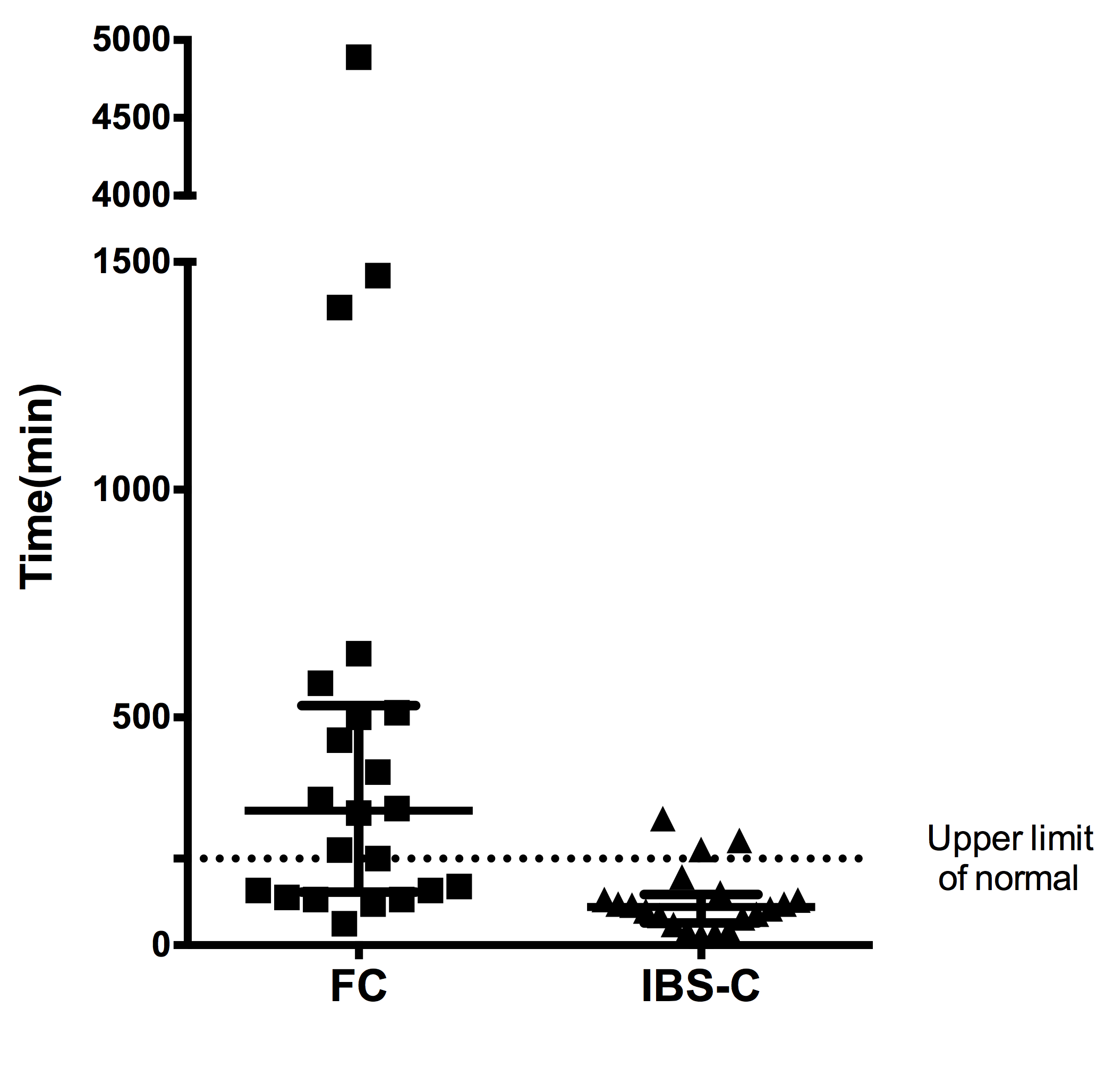
**

**Supplemental Figure 3: Time to first bowel movement (min) following ingestion of Moviprep^®^ for Functional Constipation (FC) and Irritable bowel syndrome with constipation (IBS-C) patients. Those with FC were significantly slower to respond, p<0.01, with 60% exceeding our upper limit of normal of 6 hours while only 3 IBS-C patients did, and by only a short time whereas some FC patients took several days to respond.**
